# Transcriptome analysis defines myocardium gene signatures in children with ToF and ASD and reveals disease-specific molecular reprogramming in response to surgery with cardiopulmonary bypass

**DOI:** 10.1186/s12967-020-02210-5

**Published:** 2020-01-10

**Authors:** Federica Raggi, Davide Cangelosi, Pamela Becherini, Fabiola Blengio, Martina Morini, Massimo Acquaviva, Maria Luisa Belli, Giuseppe Panizzon, Giuseppe Cervo, Luigi Varesio, Alessandra Eva, Maria Carla Bosco

**Affiliations:** 1 Laboratory of Molecular Biology, IRCSS Istituto Giannina Gaslini, Padiglione 2, L.go G.Gaslini 5, 16147 Genova, Italy; 2 Department of Cardiology, IRCSS Istituto Giannina Gaslini, Genova, Italy; 3 Present Address: Department of Internal Medicine, University of Genova, Genova, Italy; 4 Present Address: INSERM U955 Equipe 16, Creteil, France; 5 Present Address: Immunobiology of Neurological Disorders Unit, Institute of Experimental Neurology INSPE, Ospedale San Raffaele, Milano, Italy; 6 Present Address: Cytomorphology Laboratory, Heamo-Onco-TMO Department, IRCSS Istituto Giannina Gaslini, Genova, Italy

**Keywords:** Gene expression profiling, Congenital heart disease, Cardiopulmonary bypass, Atrial myocardium, Hypoxia

## Abstract

**Background:**

Tetralogy of Fallot (ToF) and Atrial Septal Defects (ASD) are the most common types of congenital heart diseases and a major cause of childhood morbidity and mortality. Cardiopulmonary bypass (CPB) is used during corrective cardiac surgery to support circulation and heart stabilization. However, this procedure triggers systemic inflammatory and stress response and consequent increased risk of postoperative complications. The aim of this study was to define the molecular bases of ToF and ASD pathogenesis and response to CPB and identify new potential biomarkers.

**Methods:**

Comparative transcriptome analysis of right atrium specimens collected from 10 ToF and 10 ASD patients was conducted before (Pre-CPB) and after (Post-CPB) corrective surgery. Total RNA isolated from each sample was individually hybridized on Affymetrix HG-U133 Plus Array Strips containing 38,500 unique human genes. Differences in the gene expression profiles and functional enrichment/network analyses were assessed using bioinformatic tools. qRT-PCR analysis was used to validate gene modulation.

**Results:**

Pre-CPB samples showed significant differential expression of a total of 72 genes, 28 of which were overexpressed in ToF and 44 in ASD. According to Gene Ontology annotation, the mostly enriched biological processes were represented by matrix organization and cell adhesion in ToF and by muscle development and contractility in ASD specimens. GSEA highlighted the specific enrichment of hypoxia gene sets in ToF samples, pointing to a role for hypoxia in disease pathogenesis. The post-CPB myocardium exhibited significant alterations in the expression profile of genes related to transcription regulation, growth/apoptosis, inflammation, adhesion/matrix organization, and oxidative stress. Among them, only 70 were common to the two disease groups, whereas 110 and 24 were unique in ToF and ASD, respectively. Multiple functional interactions among differentially expressed gene products were predicted by network analysis. Interestingly, gene expression changes in ASD samples followed a consensus hypoxia profile.

**Conclusion:**

Our results provide a comprehensive view of gene reprogramming in right atrium tissues of ToF and ASD patients before and after CPB, defining specific molecular pathways underlying disease pathophysiology and myocardium response to CPB. These findings have potential translational value because they identify new candidate prognostic markers and targets for tailored cardioprotective post-surgical therapies.

## Background

Congenital heart diseases (CHDs) are the most frequent types of birth defects in humans, affecting over 1% of all live births worldwide (estimated incidence 8 per 1000), and represent a major cause of morbidity and mortality in children [1]. CHDs can be classified into three broad categories: cyanotic heart disease, left-sided obstructive defects, and septation defects [2]. Tetralogy of Fallot (ToF) is the main form of cyanotic CHDs (estimated incidence 5 per 10,000 live births), characterized by a conal septum malalignment which leads to aorta rightward deviation. This defect results in a large ventricular septal malformation and stenosis of the pulmonary valve with consequent pressure and volume overload of the right ventricle, adaptive ventricular hypertrophy associated with reduced pulmonary flow, impaired myocardial nutrient and oxygen supply, and finally heart failure [3]. ToF patients require primary surgical repair during the first year of life to close the ventricular defect and remove the obstruction in order to relieve hypoxemia, eliminate the hypertrophic stimulus, and preserve the function of the right ventricle. However, long-term complications and probability of secondary corrective surgery later in life remain important clinical challenges [3, 4]. Atrial Septal Defects (ASD) are the third most common types of CHD (estimated incidence 10 per 10,000 live births). They are characterized by several defects in the cardiac terminations of systemic and pulmonary veins and in the intratrial septum, which result in the communication of the heart left and right sides and blood shunt between pulmonary and systemic circulations [5]. Only a few ASD patients present with severe problems during infancy and require primary cardiac surgery within the first year of life to prevent the onset of irreversible changes in the pulmonary vasculature [6]. Most ASD patients are asymptomatic throughout infancy and childhood but may develop complications that increase with age, which include ventricle dysfunction, atrial arrhythmias, pulmonary hypertension, and heart failure. Life expectancy is reduced if defects remain untreated, recommending surgery at the age of 4/5 years [5, 6]. Surgical closure of atrial septal defects is usually associated with normal life expectancy [5].

Cardiopulmonary bypass (CPB) with aortic crossclamping (AoXC) and hypothermic cardioplegic arrest (CA) is a commonly used technique in cardiac surgery to support circulation and heart stabilization and maintain organ perfusion. It facilitates the repair of cardiac lesions resulting in the reduction of surgical mortality and achievement of complete repair of heart defects also at an early age [4, 7]. However, despite the efforts to minimize organ damage, cardiac surgery with CPB is associated with postoperative morbidity and multiorgan dysfunction syndrome. It is well documented that CPB triggers a systemic inflammatory response, whose activation in the setting of major surgery and trauma can be exaggerated in some patients, resulting in the inappropriate recruitment and hyperactivation of leukocytes (mainly neutrophils and monocyte/macrophages), increased release of proinflammatory cytokines, excessive stimulation of the complement and coagulation systems, and endothelial dysfunction, eventually leading to unwarranted organ damage [8, 9]. In addition, myocardium subjected to CA undergoes an obligate period of ischemia lasting about 1 h. Subsequent reperfusion of ischemic myocardium (I/R) causes the release of reactive oxygen species (ROS), apoptosis, and necrosis which can further aggravate CPB-induced inflammatory and stress response, contributing to organ dysfunction and increasing the risk of postoperative complications and myocardial failure [9–12].

Although significant advances in the clinical management of ToF and ASD patients have been made in the last few decades, much remains to be elucidated regarding the molecular mechanisms underlying disease pathogenesis and myocardial response to corrective surgery with CPB. In the field of cardiomyopathy, microarray-based gene expression profiling has become an important approach for the characterization of the molecular bases of disease pathogenesis, progression, and response to surgery/therapy, contributing to the identification of novel biomarkers essential for the refinement of patient diagnostic and prognostic evaluation and the design of tailored treatment strategies [10, 13–17].

In this study, we conducted a comparative transcriptome analysis of right atrium biopsies obtained from children affected by ToF and ASD undergoing primary surgical defect repair before and after CPB. Our results define disease-specific myocardial transcriptional signatures and identify distinct patterns of gene expression occurring in response to CPB in the two pathologies, providing a framework for the identification of new potential prognostic markers and targets for tailored post-surgical treatment strategies.

## Methods

### Study population

Ten patients affected by cyanotic ToF (6 males, 4 females, mean age 1 year) and ten patients affected by ASD (4 males, 6 females, mean age 5 years) undergoing primary corrective surgery with CPB at the Department of Cardiosurgery of the Gaslini Institute were enrolled in the study from July 2008 through December 2013. The surgical procedure used has been previously standardized, as reported [12]. ToF patients were classified as cyanotic according to arterial blood oxygen saturation. All patients were in stable conditions without preoperative respiratory or ionotropic support and were admitted to the intensive care unit of the Gaslini Institute after surgery. The protocol was reviewed and approved by the Ethical Committee of the Gaslini Institute prior to starting the study, and the procedures were carried out according to the approved guidelines and in adherence to the general ethical principles set forth in the Declaration of Helsinki. Written informed consent was obtained from the parents or a legally authorized representatives of the patients enrolled in the study prior to sample collection.

### Tissue sample collection

Pre- and post-operative biopsy specimens from right atrium were collected at the time of surgical defect correction. The first biopsy was harvested about 5 min before AoXC (Pre-CPB), whereas the second biopsy was harvested about 15 min after AoXC removal (Post-CPB) (average CPB duration did not exceed 70 min). Specimens were harvested with cold sharp dissection, immediately snap-frozen in liquid nitrogen, and stored until use at − 80 °C in the Integrating Tissueomics Biobank (BIT)–Gaslini, which was set up for storing tissue and genomic specimens for diagnostic and research purposes under the initial approval of the Ethical Committee of the Gaslini Institute and the subsequent ratification of the Ethical Committee of the Regione Liguria (Approval 8/2014). Atrial sampling was atraumatic, provided full-thickness specimens, and was clinically reproducible, as previously reported by Voisine et al. [13].

### RNA isolation and cRNA synthesis

Tissue specimens were mechanically homogenized in lysis reagent from Qiagen (Milano, Italy). The procedure of RNA isolation and cRNA synthesis has been previously described [18]. Briefly, total RNA was purified and DNase treated using the RNeasy MiniKit (Quigen), controlled for integrity by nanoelectrophoresis using an Agilent 2100 Bioanalyzer (Agilent Technologies Europe, Waldbroon, Germany), quantified by spectrophotometry using a NanoDrop ND-1000 (NanoDrop Technologies, Wilmington, USA), and reverse-transcribed into double-stranded cDNA on a GeneAmp PCR System 2700 thermal cycler (Applied Biosystems, Milano) using the one-cycle cDNA synthesis kit (Affymetrix, Milano). cDNA was purified, transcribed into cRNA, and biotin labeled using the GeneChip IVT kit (Affymetrix). Labeled cRNA was fragmented according to Affymetrix’s instructions.

### GeneChip hybridization and microarray data analysis

Gene expression profiling of the samples was performed by microarray analysis as detailed previously [18]. Briefly, fragmented cRNA was hybridized on Affymetrix Human Genome U133 Plus PM Array Strips (Thermo Fisher) containing 54,675 probe sets coding for 47,000 transcripts and variants, including 38,500 unique human genes on a single array. Chips were stained with streptavidin–phycoerythrin (Invitrogen Life Technologies, Milano) and scanned using an Affymetrix GeneChip Scanner 3000. Expression values were quantified, and data were processed by RMA normalization utilizing the ‘Affy’ R package. Statistical analysis using Student’s t-test was performed to identify differentially expressed probe sets. Specifically, unpaired Student’s t-test was used to assess whether there were significant differentially expressed probe sets between ASD and ToF, whereas paired Student’s t-test was used to identify differences in the probe set expression levels between Pre-CPB and Post-CPB in ASD or in ToF. Student T test was calculated by GraphPad Prism version 6.0 for Windows (http://www.graphpad.com). We corrected the p value by Benjamini- Hochberg method for false discovery rate (FDR) control. Only probe sets differences that passed the test at a FDR ≤ 0.05 were considered significant. Fold-change (FC) was calculated to determine the magnitude of the difference. Probe sets were considered significantly differentially expressed if they exhibited a FDR ≤ 0.05 and FC ≥ 2 or ≤ 0.5. We filtered out probe sets having a coefficient of variation (CV) lower than 0.7, because they did not substantially change between the two diseases under consideration, and those having an expression value lower than 100 in at least 20% of the samples because they were not sufficiently expressed in our data set to provide a reliable transcriptional level. Filtering was carried out by ‘GeneFilter’ R package, as described [18, 19]. We converted the Affymetrix probe sets into the corresponding gene symbol by Netaffix tool. When multiple probe sets were associated with the same gene symbol, the probe set with the highest expression signal was considered [19]. The full set of data from each microarray experiment has been deposited at the Gene Expression Omnibus (GEO) public repository at NCBI (http://www.ncbi.nlm.nih.gov) and can be accessed to through GEO Series accession number GSE132176. Differentially expressed genes (DEGs) were visualized by heat-map representation obtained by Morpheus heat map building tool (http://www.broadinstitute.org/cancer/software/morpheus/) available from the Broad Institute.

### Gene Ontology annotation and gene network analysis

Gene Ontology (GO) enrichment analysis of DEGs was carried out using the Cytoscape BINGO plugin [20]. DEGs were classified according to the biological process and cellular component GO collections. Terms with p value and FDR lower than 0.05 were considered significantly enriched. Correction for multiple hypothesis testing was carried out by the Benjamini–Hochberg method, as described by Maere et al. [20]. The Search Tool for the Retrieval of Interactive Gene Database (STRING-DB) Version 9.1. (http://string-db.org/) was used to construct functional interaction networks among DEGs-encoded proteins [21]. To this end, we performed a STRING-DB multiple proteins search using the DEGs as input list and extracted all the potential connection among encoded proteins. We set up a required minimum interaction score of 0.7 (high confidence) and considered significant an enrichment p-value ≤ 0.05.

### Gene set enrichment analysis

Gene Set Enrichment Analysis (GSEA) was carried out on all probe sets of the Affymetrix HG-U133 Plus 2.0 PM GeneChip microarray to evaluate the enrichment of hypoxia-related genes in ToF and ASD expression profiles, as described [18]. To this end, we built a custom gene set collection, named “HeartHypoxia”, by selecting 109 gene sets among the curated collections of the Broad Institute Molecular Signature v5 Database (MSigDB) [22]. Available gene sets were listed using “hypoxia” and “heart” as keywords. We considered gene sets containing between 15 and 500 probe sets and collapsed the expression set to gene symbol before running the analysis. An enrichment with FDR q-values ≤ 0.2 and nominal p values ≤ 0.05 was considered significant.

### Real-time RT-PCR

cDNA was prepared from purified total RNA using SuperScript Double-Stranded cDNA synthesis kit (Invitrogen). Quantitative real time PCR (qRT-PCR) was performed on a 7500 Real Time PCR System (Applied) using SYBR Green PCR Master Mix and sense/antisense oligonucleotide primers synthesized by TIBMolbiol (Genova), as previously detailed [18, 23]. Expression data were normalized on the values obtained in parallel for three reference genes (actin related protein 2/3 complex subunit 1B, ARCP1B; lysosomal-associated multispanning membrane protein-5, LAPTM5; and ribosomal protein S3, RSP3), using the Bestkeeper software, and relative expression values were calculated using Q-gene software, as described [24].

## Results

### Comparative transcriptome analysis of atrium specimens from ToF and ASD patients

**Fig. 1:**
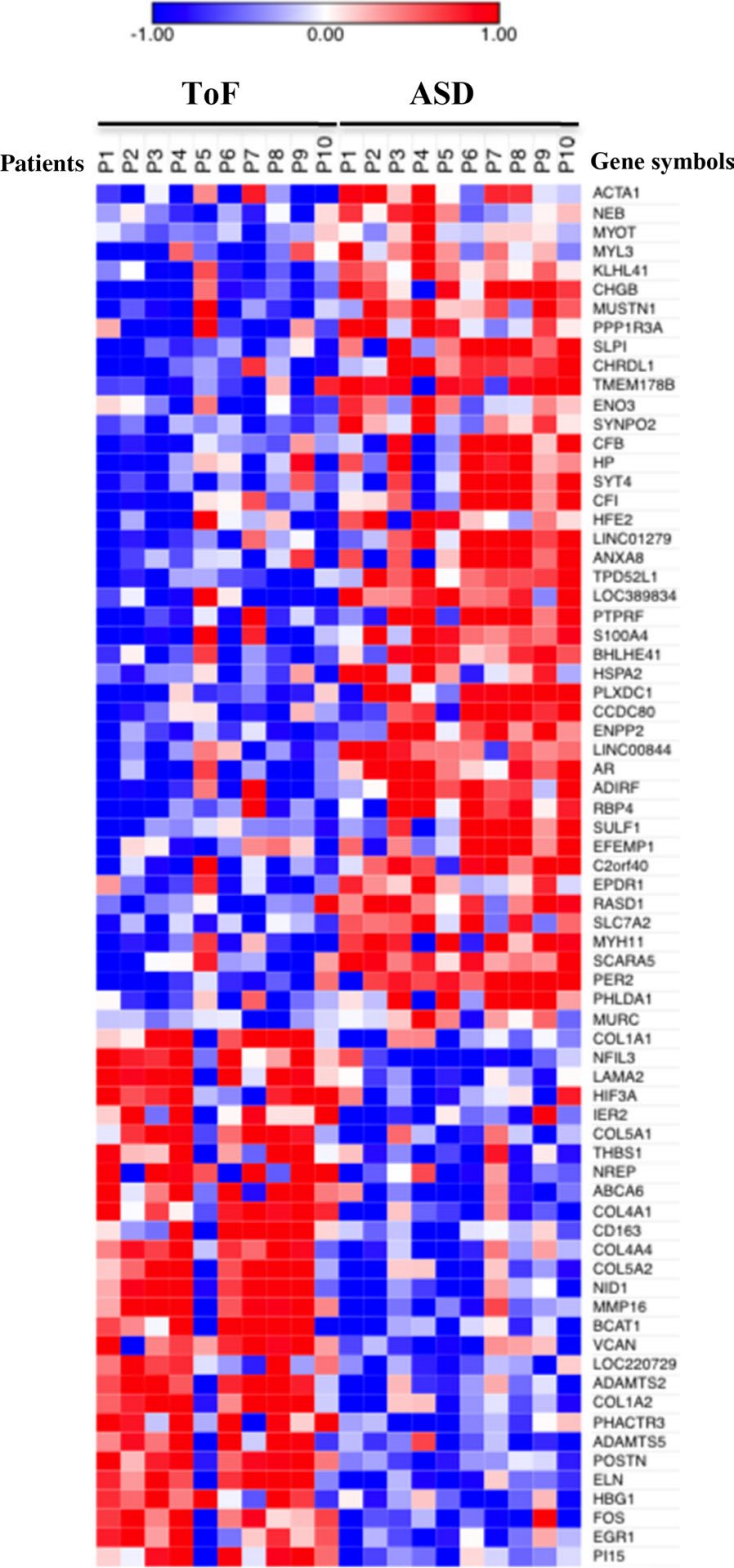
Heat-map representation of DEGs in ToF compared to ASD atrium tissues. Data shown are relative to gene expression of atrium tissues from 10 ToF and 10 ASD patients undergoing cardiac surgery profiled by microarray. The heat-map shows the expression levels of genes differentially expressed in ToF vs ASD samples. Expression levels were z-scored and log2 transformed and are indicated by a 2-color scale ranging from blue (lowest values) to red (highest values). The 2-color scale is reported in the horizontal bar at the top of the figure. Each column represents a patient and each row represents a gene. The gene symbols are listed on the right side of the heatmap, whereas the disease type is indicated on the top side

To identify genes involved in ToF and ASD pathogenesis, we compared by microarray analysis the transcriptome of right atrial specimens collected from 10 ToF and 10 ASD patients at the time of corrective surgery. cRNA derived from total RNA isolated from each sample was individually hybridized to human Affymetrix HG-U133 Plus PM Array Strips (GEO database accession number GSE132176), and raw data were processed as described in “Methods” section. Probe set expression differences of ≥ twofold and p-value ≤ 0.05 between ToF and ASD samples were considered statistically significant. Using these selection criteria, we identified a total of 89 differentially expressed probe sets (see Additional file 1: Table S1) corresponding to 72 unique DEGs in ToF vs ASD samples. Among them, 28 were overexpressed in ToF respect to ASD patients while 44 were overexpressed in ASD respect to ToF. Heat map visualization of the expression values showed a clear separation between the two diagnostic groups, with substantial homogeneity among patients affected by the same pathology (Fig. 1).

**Fig. 2:**
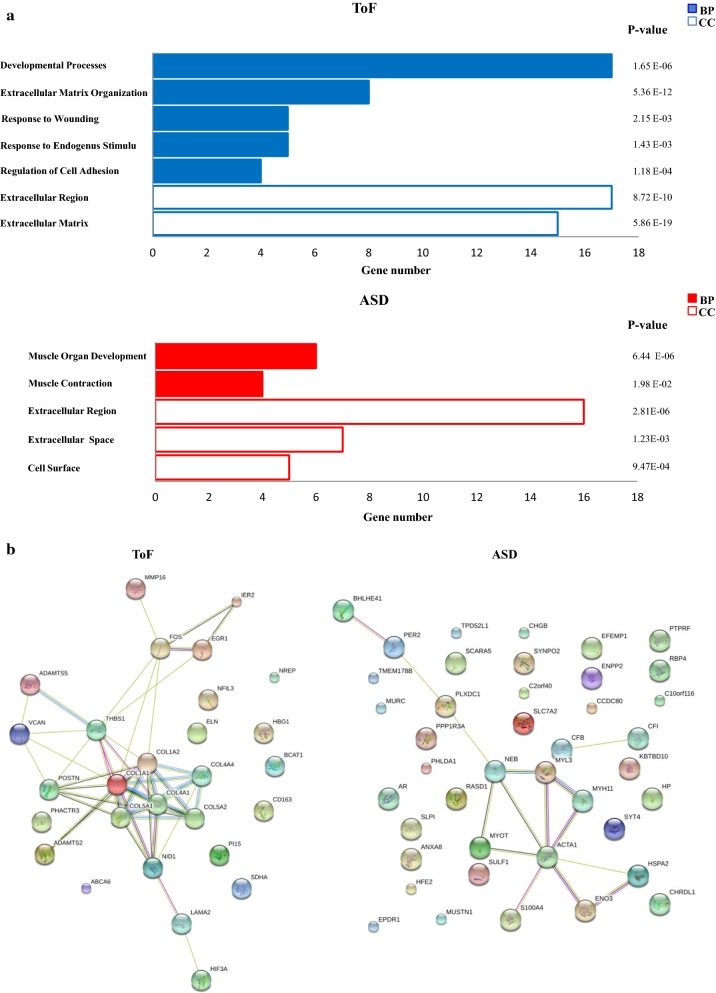
Functional enrichment and network analyses of DEGs in ToF vs ASD samples. **a** GO enrichment analysis. Genes showing at least twofold expression differences between ToF and ADS specimens were analyzed using the GO biological process (BP, *full columns*) and cellular component (CC, *empty columns*) collections. A GO term was significantly enriched if p-value ≤ 0.05 and FDR ≤ 0.05. Genes could be comprised in more than one term depending on the function of the encoded protein. The graph shows the most relevant GO terms. The GO term name is reported on the *y*-*axis*; the number of enriched DEGs for each term is indicated on the *x*-*axis*. GO terms are listed by decreasing number of DEGs. The p value for each GO term is indicated. **b** Network analysis. The STRING-DB software was used to construct functional interaction networks among DEGs products. Networks are displayed graphically as nodes (DEGs products) and edges (predicted protein–protein associations). Colored nodes indicate query DEGs and first shell of interactors. Filled nodes indicate some 3D structure known or predicted. The color of the edge indicates the type of interaction inferred by experimental evidence (*magenta lines*), text-mining (*light green lines*), curated databases (*cyan lines*), encoded-protein homology (*purple lines*), and co-expression (*violet lines*). The thickness of the line indicates the degree of confidence prediction of the association. Only associations with a high degree of confidence (0.7) are displayed in the plot

GO analysis was then carried out on the 72 DEGs to assess their biologic function. GO terms with significant enrichment score were selected. The analysis based on the biological process collection identified a total of 86 significantly enriched (p-value ≤ 0.05 and FDR ≤ 0.05) GO terms that differed between the two disease groups (70 in ToF and 16 in ASD). As depicted in Fig. 2a, the mostly enriched biologic process in ToF atrial samples was represented by developmental processes, followed by extracellular matrix (ECM) organization, response to wounding and to endogenous stimulus, and regulation of cell adhesion. Conversely, the top functional processes in ASD samples were implicated in muscle development and contraction. GO annotation for cellular components was also evaluated, identifying 37 significantly enriched GO terms (18 in ToF and 19 in ADS) among which extracellular region and extracellular matrix were dominant in ToF, whereas extracellular region, extracellular space, and cell surface were mostly enriched in ASD (Fig. 2a).

Table 1 shows a selection of DEGs in the two disease types.

**Table 1:** Relative expression of selected DEGs in ToF vs ASD specimens

Function	Gene symbol	Gene description	Fold change^a^
*ToF*			
Matrix organization and cell adhesion	ELN	Elastin	3.9
	POSTN	Periostin, osteoblast specific factor	3.6
	ADAMTS5	ADAM metallopeptidase with thrombospondin type 1 motif, 5	3.6
	COL1A1	Collagen, type I, alpha 1	3.5
	COL1A2	Collagen, type I, alpha 2	2.8
	ADAMTS2	ADAM metallopeptidase with thrombospondin type 1 motif, 2	2.8
	COL5A1	Collagen, type V, alpha 1	2.7
	VCAN	Versican	2.7
	COL5A2	Collagen, type V, alpha 2	2.6
	MMP16	Matrix metallopeptidase 16 (membrane-inserted)	2.5
	NID1	Nidogen 1	2.3
	COL4A4	Collagen, type IV, alpha 4	2.3
	COL4A1	Collagen, type IV, alpha 1	2.1
	THBS1	Thrombospondin 1	2.1
	LAMA2	Laminin, alpha 2	2.1
Trascription factors	EGR1	Early growth response 1	4.5
	FOS	FBJ murine osteosarcoma viral oncogene homolog	4.0
	HIF3A	Hypoxia inducible factor 3, alpha subunit	2.2
	IER2	Immediate early response 2	2.1
	NFIL3	Nuclear factor, interleukin 3 regulated	2.0
*ASD*			
Muscle development and contraction	ACTA1	Actin, alpha 1, skeletal muscle	9.6
	NEB	Nebulin	7.9
	MYOT	Myotilin	6.4
	MYL3	Myosin, light chain 3, alkali; ventricular, skeletal, slow	5.3
	KLHL41	Kelch-like family member 41	4.8
	MUSTN1	Musculoskeletal, embryonic nuclear protein 1	4.1
	PPP1R3A	Protein phosphatase 1, regulatory subunit 3A	3.9
	ENO3	Enolase 3 (beta, muscle)	3.6
	SYNPO2	Synaptopodin 2	3.4
	MYH11	Myosin, heavy chain 11, smooth muscle	2.0
	MURC	Muscle-related coiled-coil protein	2.0

Comparative analysis of gene expression in ToF and ASD samples was conducted as described in “Methods” section. A function, a gene symbol, a brief gene description, and the fold change values are specified for each gene

Genes for each functional category are ordered according to decreasing fold changes

^a^Fold change ≥ 2 are considered significant

Among them, genes coding for different types of collagen, namely COL1A1, COL1A2, COL5A1, COL5A2, COL4A1, and COL4A4, and matrix metalloproteinases (MMPs), such as ADAMTS5, ADAMTS2, and MMP16, were significantly upregulated in ToF samples. ToF profile also revealed significant increased expression of genes with transcription regulatory activity, such as EGR1, FOS, and HIF-3α. Functional interactions among DEGs-encoded proteins involved in matrix organization/cell adhesion and transcription regulation were predicted by network analysis (Fig. 2b). In contrast, the most relevant upregulated genes in ASD were those coding for constituents of the muscle contractile apparatus such as ACTA1, MYOT, MYL3, MUSTN1, MYH11, NEB, MURC, and ENO3 (Table 1), whose functional interactions were predicted by network analysis (Fig. 2b).

To confirm differential gene expression in the two pathologies, mRNA levels of a representative gene, EGR1, were quantified by qRT-PCR in 6 ToF and 6 ASD samples analyzed by microarray. This gene was chosen based on its known association with hypoxia and role in myocardial injury [23, 25]. As shown in the Additional file 2: Figure S1A, qRT-PCR confirmed EGR1 overexpression in ToF respect to ASD patients. The extent of modulation was higher according to qRT-PCR respect to Affymetrix data, in agreement with previous findings showing that microarray can often underestimate the degree of gene regulation [23].

Previous evidence demonstrated chronic exposure to hypoxia in cyanotic ToF patients [26]. GSEA was applied to determine the contribution of hypoxia to ToF myocardial transcriptome. One hundred nine gene sets belonging to all curated collections of the MSigDB v5 database were selected utilizing “hypoxia” and “heart” as keywords. Fourteen hypoxia-related gene sets were significantly enriched (FDR q-value ≤ 0.2 and nominal p-value ≤ 0.05) in the transcriptional profile of atrial tissues from ToF compared to ASD patients (Table 2), whereas none was found significantly enriched in the ASD compared to ToF transcriptome (data not shown).

**Table 2:** Hypoxia-related gene sets enriched in ToF vs ASD samples

GSEA term^a^	NOM p-val^b^	FDR q-val^c^
BIOCARTA_AMI_PATHWAY	0.00	0.00
FARDIN_HYPOXIA_11	0.00	0.03
IKEDA_MIR30_TARGETS_DN	0.02	0.09
QI_HYPOXIA	0.00	0.14
MAINA_VHL_TARGETS_DN	0.01	0.16
KIM_HYPOXIA	0.02	0.16
MENSE_HYPOXIA_UP	0.02	0.17
ELVIDGE_HYPOXIA_BY_DMOG_UP	0.05	0.18
MANALO_HYPOXIA_UP	0.02	0.18
ELVIDGE_HIF1A_AND_HIF2A_TARGETS_DN	0.01	0.19
JIANG_HYPOXIA_VIA_VHL	0.04	0.19
HARRIS_HYPOXIA	0.04	0.19
ELVIDGE_HIF1A_TARGETS_DN	0.00	0.19
LEONARD_HYPOXIA	0.04	0.20

Gene sets are ordered according to increasing FDR q-val

^a^Gene sets enriched in the GSEA analysis. Gene sets belonging to all curated collections of the MSigDB were selected using the keywords “hypoxia” and “heart” and filtering out those having less than 15 probe sets and more than 500

^b^NOM p-val measures the statistical significance of the normalized enriched score by an empirical permutation test using 1.000 gene permutations. Values ≤ 0.05 are considered significant

^c^FDR q-value is the estimated probability that the normalized enrichment score represents a false positive finding. Values ≤ 0.2 are considered significant

**Fig. 3:**
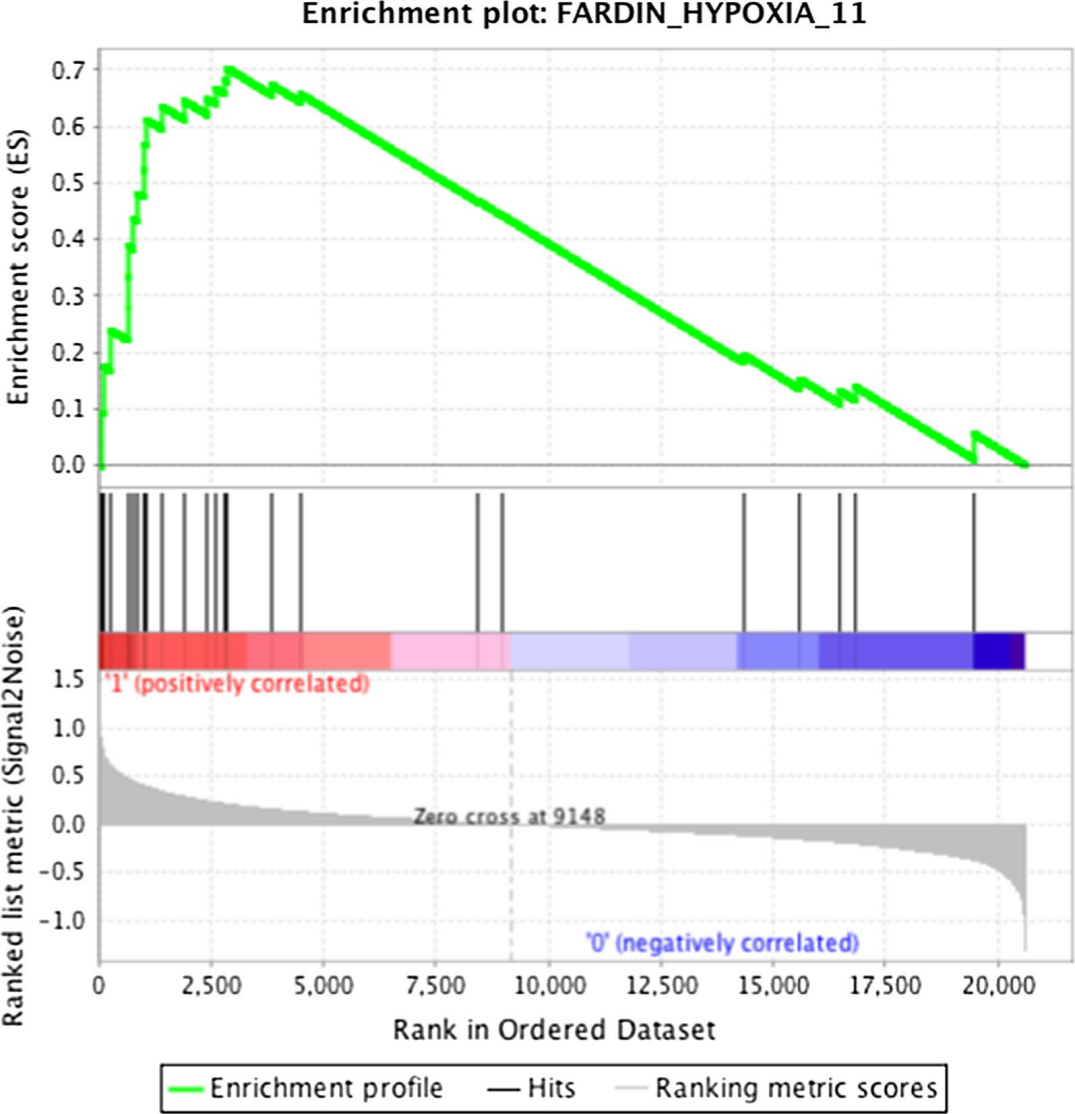
GSEA enrichment plot for the “FARDIN_HYPOXIA_11” gene set in the ToF transcriptome. The ranked list of genes identified by microarray analysis between the ToF and the ASD transcriptomes was compared with previously published gene sets of hypoxia-regulated genes by GSEA. Enrichment plot of the “FARDIN_HYPOXIA_11” gene set is shown. Signal2noise was used as metric to generate the ranked gene list

A representative enrichment plot, “FARDIN_HYPOXIA_11” [27], showing a clear enrichment of the Fardin gene set at the top of the ranked list of genes is presented in Fig. 3 for a visual inspection of the GSEA results. These data indicate that gene expression changes in ToF atrium samples follow a consensus hypoxia transcriptional profile.

Taken together, these results reveal disease-specific gene signatures in atrium samples from ToF and ASD patients, with overexpression of genes involved in myocardium remodeling and coding for hypoxia targets in the former and genes related to myocardium contractility and function in the latter.

### Gene expression changes induced by CPB in the ToF and ASD myocardium

**Fig. 4:**
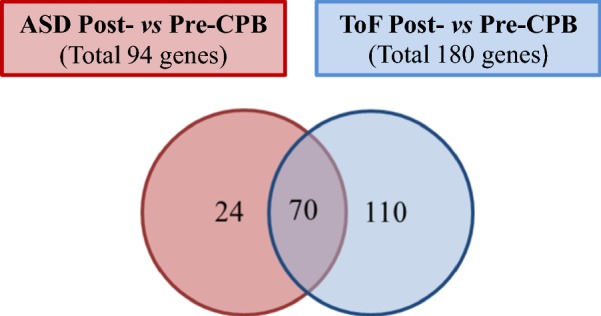
Venn diagram of DEGs induced by CPB in ToF and ASD atrium tissues. The gene expression profile of atrium tissues from 10 ToF and 10 ADS patients was compared before and after CPB, as described in “Methods” section. The diagram shows the number of common and unique DEGs in Post-CBP vs Pre-CBP samples in the two patient groups

Limited information is currently available on the molecular mechanisms mediating CPB pathogenic effects in ToF and ASD. To address this issue, we compared the transcriptional profile of right atrium tissues from ToF and ASD patients before (Pre-CPB) and after (Post-CPB) surgery. A total of 267 and 136 probe sets were identified as differentially regulated in Post- vs Pre-CPB samples from ToF and ASD patients, respectively (see Additional file 3: Table S2 and Additional file 4: Table S3), which corresponded to 180 and 94 unique DEGs. As shown by the Venn diagram in Fig. 4, 70 DEGs were commonly modulated by CPB in the two disease groups, whereas 110 and 24 were identified as specifically affected in ToF and ASD, respectively. Gene up-regulation was the predominant change that differentiated Post-CPB from Pre-CPB samples, although a large subset of genes was also found downregulated in ASD patients (Tables 3, 4, and 5; Additional file 3: Table S2 and Additional file 4: Table S3). These data indicate that CPB induces distinct cardiac gene expression changes in these two forms of CHDs.

**Table 3:** Relative expression of selected genes commonly regulated in Post- vs Pre-CPB right atrial samples from ToF and ASD patients

Function	Gene symbol	Gene description	Fold change^a^	
			ToF	ASD
Trascription regulation	ATF3	Activating transcription factor 3	35.8	13.4
	EGR3	Early growth response 3	27.0	13.0
	NR4A2	Nuclear receptor subfamily 4, group A, member 2	20.7	18.2
	NR4A3	Nuclear receptor subfamily 4, group A, member 3	19.6	4.8
	FOSB	FBJ murine osteosarcoma viral oncogene homolog B	14.9	28.8
	MAFF	v-maf avian musculoaponeurotic fibrosarcoma oncogene homolog F	12.0	8.4
	JUNB	Jun B proto-oncogene	10.3	9.4
	EGR2	Early growth response 2	9.8	5.9
	EGR1	Early growth response 1	8.8	20.5
	NR4A1	Nuclear receptor subfamily 4, group A, member 1	6.5	5.3
	NFKBIZ	Nuclear factor of kappa light polypeptide gene enhancer in B-cells inhibitor, zeta	6.3	2.2
	MYC	v-myc avian myelocytomatosis viral oncogene homolog	6.0	3.8
	FOS	FBJ murine osteosarcoma viral oncogene homolog	5.9	13.9
	JUN	Jun proto-oncogene	5.9	3.9
	KLF4	Kruppel-like factor 4 (gut)	5.7	4.5
	CSRNP1	Cysteine-serine-rich nuclear protein 1	5.6	4.8
	IER3	Immediate early response 3	4.6	2.3
	FOSL2	FOS-like antigen 2	4.5	2.8
	IER2	Immediate early response	2 4.0	5.0
	HES1	Hes family bHLH transcription factor 1	3.9	2.2
	NFIL3	Nuclear factor, interleukin 3 regulated	3.9	2.4
	KLF2	Kruppel-like factor 2	2.3	2.2
Regulation of cell growth and apoptosis	MIR21/VMP1	MicroRNA 21/vacuole membrane protein 1	11.4	5
	DUSP5	Dual specificity phosphatase 5	5.4	3.1
	GADD45B	Growth arrest and DNA-damage-inducible, beta	5.2	2.6
	CDKN1A	Cyclin-dependent kinase inhibitor 1A (p21, Cip1)	5.1	2.7
	MIR22/MIR22HG	MicroRNA 22/MIR22 host gene	4.5	2.8
	MCL1	Myeloid cell leukemia 1	4.4	2.5
	MIR23A/MIR24-2	MicroRNA 23a/24-2	4.3	3.3
	BTG2	BTG family, member 2	4.0	2.6
	DUSP6	Dual specificity phosphatase 6	3.7	3.2
	CCNL1	Cyclin L1	3.7	2
	PPP1R15A/GADD34	Protein phosphatase 1, regulatory subunit 15A/growth arrest and DNA-damage-inducible	3.3	2.1
	DUSP1	Dual specificity phosphatase 1	2.2	2.1
Inflammatory response	SOCS3	Suppressor of cytokine signaling 3	23.0	24.6
	PTGS2	Prostaglandin-endoperoxide synthase 2 (prostaglandin G/H synthase and cyclooxygenase)	12.2	6.9
	CCL2	Chemokine (C–C motif) ligand 2	10.4	6.2
	CXCL2	Chemokine (C–X–C motif) ligand 2	9.3	11.7
	RGS1	Regulator of G-protein signaling 1	4.0	4.8
	RGS2	Regulator of G-protein signaling 2	3.6	2.0
	C3	Complement component 3	0.4	0.3
	ITLN1	Intelectin 1 (galactofuranose binding)	0.05	0.1
Cell adhesion and matrix organization	ADAMTS1	ADAM metallopeptidase with thrombospondin type 1 motif, 1	6.5	3.8
	CYR61 (CCN1)	Cysteine-rich, angiogenic inducer, 61	5.5	5.2
	THBD	Thrombomodulin	4.6	2.4
	EFEMP1	EGF containing fibulin-like extracellular matrix protein 1	0.4	0.4
	COL3A1	Collagen, type III, alpha 1	0.2	0.2
Antioxidant activity	MT1M	Metallothionein 1M	14.3	5.9
	NCOA7	Nuclear receptor coactivator 7	4.7	2.2
	MT2A	Metallothionein 2A	3.1	2.0

Comparative analysis of gene expression in Pre-CPB and Post-CPB ToF and ASD samples was conducted as described in “Methods” section. A function, a gene symbol, a brief gene description, and fold change values are specified for each gene

Genes for each functional category are ordered according to decreasing fold changes of ToF genes

^a^Fold change was calculated as the ratio between the mean expression value of Post-CPB and Pre-CPB samples for each gene. Fold change ≥ 2 or ≤ 0.5 are considered significant

**Table 4:** Relative expression of genes selectively modulated in Post- vs Pre-CPB ToF samples

Function	Gene symbol	Gene description	Fold change^a^
Transcription regulation	IRF1	Interferon regulatory factor 1	10.3
	BHLHE40	Basic helix-loop-helix family, member e40	4.4
	MIR612/NEAT1	MicroRNA 612/ nuclear paraspeckle assembly transcript 1	3.7
	KLF10	Kruppel-like factor 10	3.1
	BCL3/MIR8085	B-cell CLL/lymphoma 3/microRNA 8085	3.1
	IFRD1	Interferon-related developmental regulator 1	2.9
	DDIT3	DNA-damage-inducible transcript 3	2.7
	SOX9	SRY (sex determining region Y)-box 9	2.5
	ETS2	v-ets avian erythroblastosis virus E26 oncogene homolog 2	2.4
	CEBPB	CCAAT/enhancer binding protein (C/EBP), beta	2.1
	CEBPD	CCAAT/enhancer binding protein (C/EBP), delta	2.0
	AFF4	AF4/FMR2 family, member 4	2.0
Inflammatory responses	CXCL8	Chemokine (C-X-C motif) ligand 8	19.6
	IL6	Interleukin 6	18.7
	AREG	Amphiregulin	10.1
	TNFAIP3	Tumor necrosis factor, alpha-induced protein 3	3.0
	IL1RL1	Interleukin 1 receptor-like 1	2.8
	SQSTM1	Sequestosome 1	2.7
	HSPA1A,1B	Heat shock 70kDa protein 1A,1B	2.2
	KDM6B	Lysine (K)-specific demethylase 6B	2.0
	C1QC	Complement component 1, q subcomponent, C chain	0.5
	C1QB	Complement component 1, q subcomponent, B chain	0.4
Cell adhesion, cytoskeleton and matrix organization	ABRA	Actin-binding Rho activating protein	8.5
	THBS1	Thrombospondin 1	7.9
	PDLIM3	PDZ and LIM domain 3	5.3
	MMP19	Matrix metallopeptidase 19	4.6
	FBN2	Fibrillin 2	2 4
	XIRP1 (CMYA1)	Xin actin-binding repeat containing 1	3.6
	HBEGF	Heparin-binding EGF-like growth factor	2.6
	CTNNB1	Catenin (cadherin-associated protein), beta 1, 88 kDa	2
Antioxidant activity	MT1X	Metallothionein 1X	3.9
	MT1F	Metallothionein 1F	3.1
	MT1HL1	Metallothionein 1H-like 1	3.1
	MT1H	Metallothionein 1H	2.9
	MT1E	Metallothionein 1E	2.9
	MT1G	Metallothionein 1G	2.5
DNA repair	SPIDR	Scaffolding protein involved in DNA repair	5.5
	PPP1R10	Protein phosphatase 1, regulatory subunit 10	2.1

Comparative analysis of gene expression in Pre-CPB and Post-CPB ToF samples was conducted as described in “Methods” section. A main function, a gene symbol, a brief gene description, and the fold change values are specified for each gene

Genes for each functional category are ordered according to decreasing fold changes

^a^Fold change was calculated as the ratio between the mean expression value of Post-ToF and Pre-ToF samples for each gene. Fold changes ≥ 2 or ≤ 0.5 are considered significant

**Table 5:** Relative expression of genes selectively modulated in Post- vs Pre-CPB ASD samples

Function	Gene symbol	Gene description	Fold change^a^
Inflammatory response	KDR (FLK1, VEGFR)	Kinase insert domain receptor (type III receptor tyrosine kinase), VEGF receptor	0.5
	C4A/C4B	Complement component 4A (Rodgers blood group)/4B (Chido blood group)	0.4
	CFIv	Complement factor I	0.3
	CFB	Complement factor B	0.2
	SLPI	Secretory leukocyte peptidase inhibitor	0.2
Cell adhesion	PTPRF	Protein tyrosine phosphatase, receptor type, F	0.4
	FLRT3	Fibronectin leucine rich transmembrane protein 3	0.2
	MSLN	Mesothelin	0.16
	PRG4	Proteoglycan 4	0.13
Oxidative stress	PTGIS	Prostaglandin I2 (prostacyclin) synthase	0.5
	AOX1	Aldehyde oxidase 1	0.5

Comparative analysis of gene expression in Pre-CPB and Post-CPB ToF samples was conducted as described in “Methods” section. A main function, a gene symbol, a brief gene description, and the fold change values are specified for each gene

Genes for each functional category are ordered according to decreasing fold changes

^a^Fold change was calculated as the ratio between the mean expression value of Post-ToF and Pre-ToF samples for each gene. Fold changes ≥ 2 or ≤ 0.5 are considered significant

**Fig. 5:**
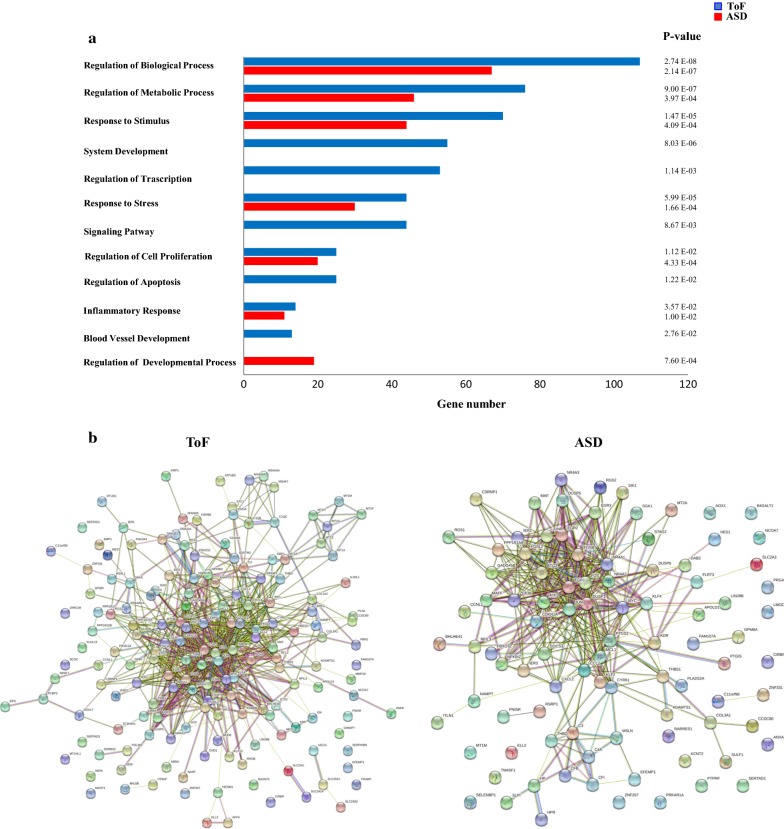
DEGs functional enrichment/network analyses in Post-CPB vs Pre-CPB atrial samples from ToF and ASD patients. **a** GO enrichment analysis. Genes showing at least twofold expression changes between Post-CPB and Pre-CPB conditions were analyzed using the GO biological processes collection, as detailed in the legend of Fig. 2a. The graph shows the most relevant GO terms. The GO term name is reported on the *y*-*axis*; the number of enriched DEGs for each term is indicated on the *x*-*axis*. The *blue columns* represent DEGs modulated in ToF patients; the *red columns* represent DEGs modulated in ASD patients. GO terms are listed by decreasing number of DEGs in ToF samples. The p value for each GO term is indicated. **b** Network analysis. Functional interaction networks among DEGs products were constructed using the STRING-DB software as detailed in the legend of Fig. 2b. Networks are displayed graphically as nodes (DEGs products) and edges (predicted protein–protein associations). Only associations with a high degree of confidence (0.7) are displayed in the plot

To gain insights into the functional processes modulated by CPB, DEGs were then analyzed by GO based on the biological process collection. We identified 61 significantly enriched processes in Post-CPB compared to Pre-CBP samples, of which 46 in ToF and 15 in ASD. Figure 5a shows a selection of the functional terms with the most significant enrichment score, the majority of which was represented in both ToF and ASD samples. Regulation of biologic and metabolic processes and response to stimulus were the top terms in post-CPB specimens from both groups of patients. The post-CPB transcriptional profile was also highly related to response to stress, regulation of cell proliferation, and inflammatory response, being a significant proportion of modulated genes significantly enriched in these processes in both disease states. The number of genes within all common processes was lower in ASD respect to ToF specimens. System development, regulation of transcription, and signaling pathways emerged as the main functional processes enriched exclusively in the Post-CPB ToF transcriptome, whereas Post-CPB ASD samples displayed specific enrichment of genes functionally implicated in the regulation of development process. Specific analysis of the 70 gene set common to both pathologies showed significant GO term enrichment in Post-CPB compared to Pre-CBP samples, most of which have been already identified by the analysis of all DEGs. In addition, we found a few terms, namely negative regulation of cell communication (p 5.76E−04), organ morphogenesis (p 4.22E−04), embryonic development (p 5.55E−05), and regulation of cytokine production (p 1.94E−03), specifically enriched in the common gene set (data not shown), indicating a substantial, but not complete, concordance between the analysis performed on the common and all DEGs after CPB. Multiple functional interactions among DEGs-encoded proteins were predicted by network analysis (Fig. 5b).

A list of the most significantly commonly regulated genes is presented in Table 3.

Among them, we found genes coding for various transcription factor family members, such as ATF, JUN, JUNB, FOS, FOSL2, NR4A1, 2, 3, EGR1,2, and 3, and IER2,3, that were upregulated in response to CPB, with the highest changes observed in the ToF group. Increased expression of genes coding for molecules with a primary role in cell proliferation and apoptosis, including CDKN1A, CCNL1, GADD45B and 34, BTG2, DUSP1, DUSP5, DUSP6, MCL1, and microRNAs 21, 22, and 23A, was also shared by the two disease groups following CPB. Another important set of genes increased in Post-CPB samples from both ToF and ASD patients coded for proinflammatory and chemotactic mediators (SOCS3, PTGS2, CCL2, CXCL2, RGS1, RGS2) and for molecules with metalloprotease (CYR61, ADAMTS1) and antioxidant activity (MT1M, MT2A). Only a few genes involved in inflammatory responses and matrix organization, namely C3, ITNL1, EFEMP1, and COL3A1, showed decreased expression in response to CPB in both disease groups.

Of the 110 genes specifically modulated by CBP in the ToF myocardium, the majority was upregulated and coded for additional regulators of transcription (such as IRF1, BHLHE40, DDIT3, ETS2, CEBPB, KLF10, BCL3, SOX9), inflammation (namely CXCL8, IL6, AREG, TNFAIP3, IL1RL1, and HSPA1A,1B), anti-oxidant response (such as MT1X, MT1F, MT1HL1, MT1H, MT1E, MT1G), cell adhesion, cytoskeleton and matrix organization (including ABRA, THBS1, XIRP1, HBEGF, and MMP19) (Table 4).

The complement component-coding genes, 1QB and 1QC, were the only genes selectively downregulated in Post-CPB ToF samples (Table 4). Conversely, post-CPB ASD samples were characterized by the specific downregulation of several genes, the most relevant of which were those involved in the regulation of the complement system (C4a,/C4b, CFI, CFB) and inflammation (VEGFR, SLP1), cell adhesion (PRG4, MSLN), and oxidative stress (PTGIS and AOX1) (Table 5).

To validate gene regulation in response to CPB, we analyzed by qRT-PCR a subset of genes upregulated in the two disease groups, selected because of their critical role as mediators of myocardial inflammatory damage [28, 29], and determined their expression levels in Post-CPB respect to pre-CPB samples from 3 ToF and 3 ASD patients analyzed by microarray. As depicted in Additional file 2: Figure S1B, we found a 100% concordance between qRT-PCR and Affymetrix data with respect to the direction of the expression changes, with the magnitude of gene induction higher in qRT-PCR than microarray data.

The myocardium subjected to CPB undergoes an obligate period of ischemia [10, 11]. We were, thus, interested to investigate the contribution of hypoxia to the post-CPB transcriptome. To this aim, we used GSEA to determine the enrichment of hypoxia gene sets in the transcriptional profile of Post-CPB specimens. As shown in Table 6, eleven hypoxia gene sets were specifically enriched in the ASD transcriptome (FDR q-value ≤ 0.2 and nominal p ≤ 0.05), whereas none was enriched in the ToF transcriptome (data non shown).

**Table 6:** Hypoxia-related gene sets enriched in Post- vs Pre-CPB ASD samples

GSEA term^a^	NOM p-val^b^	FDR q-val^c^
GROSS_ELK3_TARGETS_DN	0.002	0.012
CHEN_LVAD_SUPPORT_OF_FAILING_HEART_UP	0.018	0.014
BIOCARTA_CARDIACEGF_PATHWAY	0.002	0.014
LEONARD_HYPOXIA	0.002	0.011
GROSS_HYPOXIA_VIA_ELK3_DN	0.026	0.030
GROSS_HYPOXIA_VIA_ELK3_ONLY_UP	0.016	0.043
MENSE_HYPOXIA_UP	0.012	0.042
KRIEG_HYPOXIA_VIA_KDM3A	0.042	0.095
HARRIS_HYPOXIA	0.053	0.096
HU_ANGIOGENESIS_UP	0.048	0.101
KIM_HYPOXIA	0.050	0.142

Gene sets are ordered according to increasing FDR q-val

^a^Gene sets enriched in the GSEA analysis. Gene sets belonging to all curated collections o f the MSigDB were selected using the keywords “hypoxia” and “heart” and filtering out those having less than 15 probe sets and more than 500 probe sets

^b^NOM p-val measures the statistical significance of the normalized enriched score by an empirical permutation test using 1.000 gene permutations. NOM p-val ≤ 0.05 are considered significant

^c^FDR q-value is the estimated probability that the normalized enrichment s core represents a false positive finding. Values ≤ 0.2 are considered significant

These results indicate that CPB causes profound alterations in the myocardial transcriptome of ToF and ASD patients, modulating both shared and distinct gene sets, and suggest a reprogramming response to CPB-induced acute ischemia specifically in the ASD myocardium.

## Discussion

In the last years, several studies have demonstrated the value of myocardium transcriptome analysis for elucidating the molecular mechanisms underlying cardiovascular disease pathogenesis and identifying novel biomarkers for prognostic and therapeutic purposes [10, 13–17, 30]. In this study, we carry out the first comparative gene expression profiling of ToF and ASD right atrial specimens before and after surgery with CPB. Our results define disease-specific myocardial transcriptional signatures and demonstrate distinct molecular reprogramming in response to CPB in the two pathologies through the modulation of both common and unique genes involved in myocardial damage, inflammatory response, and oxidative stress.

ToF and ASD patients could be clearly separated into two groups based on the differential expression of 72 genes, among which 28 were specifically upregulated in ToF and 44 in ASD atrial tissues. DEGs mainly coded for extracellular proteins in both disease groups, but were involved in distinct functional processes, suggesting specific adaptive transcriptional response of the heart to the defect. ToF signature displayed enrichment of genes encoding proteins involved in ECM organization and regulation of cell adhesion, whereas ASD transcriptome was characterized by the enrichment of genes coding for proteins implicated in myocardium development and contractility.

Excessive production and deposition of ECM components represent key events in adverse cardiac remodeling, leading to fibrosis, hypertrophy, and loss of function [31]. It is, thus, conceivable that abnormal expression of genes coding for various collagen types observed in ToF atrial tissues may play an important role in disease pathogenesis. Our results are consistent with and extend previous observations showing COL1A2 and COL3A1 gene upregulation in right ventricular biopsies from ToF patients compared to age-matched controls [15]. Increased COL1A1 gene expression was also reported in cyanotic vs acyanotic ToF right ventricles [32]. Of relevance is also our evidence that MMP-coding genes were upregulated in ToF right atrium samples, given the essential contribution of ECM-degrading enzymes to cardiac remodeling and the correlation of their overexpression with the failing myocardium [33]. Interestingly, MMP gene expression has been previously shown to remain unchanged or decrease in ToF right ventricles [15]. These conflicting results could be explained by the different types of specimens analyzed in the two studies (atrium vs ventriculum) and origin of control samples used for comparison (ASD patients vs healthy children).

Previous reports have demonstrated induction of ECM proteins and MMPs by hypoxia [34, 35], a common hallmark of pathologic conditions including cardiovascular disorders [34, 36, 37], and hypoxia was suggested to affect gene expression in the ToF myocardium. Dysregulation of genes involved in oxidative phosphorylation and energy metabolism, which is a general response to conditions of reduced oxygenation [23, 36], was in fact reported in right ventricular specimens from cyanotic ToF children compared to age-matched controls [38], and upregulation of tissue remodeling genes was demonstrated in the cyanotic respect to the acyanotic ToF myocardium [26, 32]. Hence, the observed increase in collagen and MMP genes in ToF respect to ASD could probably be ascribed to the chronic hypoxic environment of the ToF myocardium. In line with this conclusion is the finding that ToF samples exhibited significant enrichment of several hypoxia-related gene sets, including genes encoding the known hypoxia transcription factors, EGR1, FOS, and HIF-3α, whose overexpression has been previously implicated in cardiac ECM remodeling, myocardial I/R, and hypertrophy [23, 25, 36, 39] and whose functional interaction with collagen- and MMP-coding genes was predicted by network analysis. Taken together, these data provide novel mechanistic evidence of the contribution of the cardiac chronic hypoxic state to ToF pathogenesis. On the basis of published findings suggesting age-associated changes in the expression of genes encoding molecules associated with extracellular matrix, cytoskeletal structure, collagen deposition, adhesion, and transcription in both mice and human hearts [40–43], it is possible that the observed gene alterations in ToF respect to ASD patients may result from a combined effect of disease state and lower patient age. Further large-scale studies are needed to specifically address this issue.

Another significant difference between ToF and ASD transcriptomes was related to the enrichment of sarcomeric protein-coding genes in ASD atrial samples. Although only MYL3 dysregulation has been previously linked to ASD pathogenesis [16], ACTA1 and MURC overexpression was reported to contribute to cardiac contractile dysfunction and conduction disturbances in mouse models [44, 45], whereas MYL3, MURC, and MYH11 gene mutations or copy number variants were found associated with cardiomyopathy or CHDs [46–48]. Based on this evidence, we hypothesize that the observed upregulation of contractile fiber genes in the ASD myocardium and functional interactions among their products, predicted by network analysis, are critical for disease development.

It was documented that the use of CPB during cardiac surgery can cause both peripheral blood and cardiac gene expression pattern alterations [10, 13, 14, 49], and modulation of ventricular transcriptome has been reported in ToF by Ghorbel et al. [26, 30]. However, no data are available on CPB effects on the gene expression profile of the ASD myocardium or of ToF atrial tissues. We found significant gene expression changes in Post- vs Pre-CPB right atrial samples from both ToF and ASD patients, that were qualitatively and/or quantitatively different between the two groups suggesting that both common and distinct molecular mechanisms may underlie CPB effects in the two pathologies. Importantly, several of the identified genes have never been associated with CPB. A high degree of interactions among dysregulated gene products could be predicted, indicating that CPB affected important functional networks. Interestingly, we observed selective enrichment of hypoxia-related gene sets in the ASD transcriptome following CPB, suggesting the specific contribution of CPB-induced acute ischemia to gene reprogramming in the ASD myocardium.

A high number of genes up-regulated by CPB in ToF and ASD coded for early regulators of transcription, the majority of which have been previously implicated in various cardiovascular pathological processes [25, 50] and found increased in Post vs Pre-CPB cardiac tissues from adult patients [13, 14, 30]. Microarray results also revealed common induction of transcription factor-coding genes whose modulation in response to CPB has not been described, among which IER2, IER3, and FOSL2 play critical role in cardiac remodeling and apopotosis, myocardial dysfunction, and heart failure [39, 51]. In addition, several transcription factor-coding genes were upregulated by CPB selectively in ToF samples, including IRF1, a member of the interferon signaling pathway with a central role in the regulation of cardiac remodeling and the induction of heart hypertrophy, fibrosis, and dysfunction [52] and proposed as a candidate biomarker in ischemic cardiomyopathy [53]. Our findings are in line with previous evidence showing increased IRF1 expression in Post-CPB biopsies from adult patients [13]. CPB also selectively induced in ToF specimens KLF10, Bcl3, SOX9, and CEBPB genes, whose expression changes have been implicated in hypertrophic and ischenic cardiomyopathies [54–57], but have never been documented in response to CPB. Collectively, these findings highlight CPB-dependent activation of both shared and unique transcription pathways involved in myocardial damage in patients affected by different CHDs.

Increased circulating levels of several proinflammatory cytokines and chemokines have been detected in the early post-CPB phase of patients undergoing heart surgery [8, 9, 28, 58] and were suggested to contribute to post-CPB systemic inflammatory response syndrome and multiorgan damage and to represent potential markers of early postoperative morbidity [28, 30, 58]. Interestingly, the myocardium was identified as a major source of cytokines/chemokines in patients with ischemic heart disease after CPB [13, 30]. We provide the first evidence that CCL2 and CXCL2 genes were upregulated in Post-CPB atrial tissues from both ToF and ASD patients, whereas only the ToF myocardium exhibited increased expression of IL-6 and CXCL8 genes after CPB. These data are intriguing and suggest that myocardial cytokine/chemokine gene expression may be differentially affected by CPB in distinct CHDs. Because the association of hypoxic preconditioning and oxygen stress is critical for CXCL8 and IL-6 gene regulation [10, 59], it is conceivable that their induction in ToF is related to the preoperative chronic ischemic state of the myocardium which increases susceptibility to the effects of reoxygenation that follows CPB.

TOFs and ASD post-CPB specimens also shared the upregulation of other genes with proinflammatory properties, such as SOCS3 and PTGS2, which encode important regulators of inflammation and may represent key mediators of myocardial cell damage [29, 60]. Our findings extend previous evidence showing SOCS3 and PTGS2 upregulation in the left ventricles of adult ischemic hearts after CPB [30] and suggest their potential contribution to myocardial inflammatory injury triggered by CPB in ToF and ASD. Increased expression of AREG gene in the ToF myocardium after CPB is also of note, given the role of the encoded protein in inflammation, tissue remodeling, and fibrosis [61]. These results suggest the potential therapeutic efficacy of targeting these genes as a cardioprotective strategy in ToF and ASD patients undergoing CPB.

Among genes linked to inflammation, we observed downregulation of those coding for C3, ITNL1, and SLP1, following CPB in ToF and/or ASD. Complement C3 was recently shown to contribute to myocardial function preservation and regeneration in a mouse model of chronic myocardial infarction [62]. The ITLN1-encoded circulating protein, Omentin 1, has a recognized role in cardiovascular disease as a “protective adipokine” able to ameliorate heart damage and function in patients with acute myocardial infarction and in mice models of I/R injury [63]. SLP1 is a potent secreted inhibitor of neutrophil proteases and recruitment, shown to contribute to the recovery of post-ischemic myocardium function [64]. ITLN1 and SLP1 products were identified as promising candidates for treatment/prevention of I/R injury and post-ischemic inflammation [63, 64]. Based on these evidences, we can hypothesize that decreased C3, ITLN1, and SLPI gene expression may contribute to CPB-induced myocardium damage in ToF and ASD patients. Our findings support previous data showing downregulation of these genes in cardiac transcriptome of patients undergoing aortic valve replacement with CPB [49], thus warranting further investigation.

The complexity of myocardial response to CPB is emphasized by the overexpression of several genes encoding cardioprotective factors, such as HSPA1A,1B, RGS2, IL1RL1, and TNFAIP3, among which only HSPA1A,1B gene up-regulation was reported in earlier studies [65]. HSPA1A,1B encodes the inducible HSP-70i isoform, whose role in myocardium protection against I/R injury was demonstrated in transgenic mice [66] and confirmed in cyanotic ToF patients [67]. The G protein receptor regulator, RGS2, has received increasing interest as a potential therapeutic target in cardiovascular disease given to its strong cardioprotective effects observed in preclinical mouse models [68]. IL1RL1 codes for the receptor of IL-33, an important biomarker of myocardial stress, fibrosis, and chronic heart failure secreted in response to cell damage. Interaction between IL-33 and IL1RL1 in experimental models results in the reduction of myocardial fibrosis and apoptosis and the improvement of cardiac function [69]. TNFAIP3 encodes an anti-inflammatory protein whose overexpression in the heart was shown to attenuate myocardial hypertrophic response and post-infarction remodeling and inflammation in transgenic mouse models, improving cardiac function [70]. Our finding extend the list of genes with cardioprotective functions previously identified in response to CPB, confirming that pro-inflammatory and cardioprotective effects are highly intertwined and defining new potential markers of adaptive myocardial response to surgical stress and potential targets of postsurgical therapy in CHD patients. The higher number of cardioprotective markers detected in ToF vs ASD myocardium is probably due to its preoperative chronic ischemic state that may induce various adaptive pathways to limit tissue damage.

Consistent with the view that CPB activates cardioprotective mechanisms in the ToF and ASD myocardium is the observation that Post- respect to Pre-CBP atrial tissues expressed higher levels of genes coding for metallothionein (MT) family members, metal-binding proteins highly inducible under stress conditions and endowed with anti-oxidant activity [71]. Oxidative stress is one of the main causes of myocardial I/R injury, and reduction of ROS generated upon I/R challenge represents an important mechanism conferring cardiac cell protection from oxidative stress [72]. MT genes upregulation may thus represent an important cardioprotective mechanism against CPB-induced oxidative stress, in agreement with previous studies in mouse models of I/R myocardial injury [73]. Upregulation of a higher number of MT genes in ToF than ASD samples indicates a positive correlation between preoperative hypoxia and expression of antioxidant genes.

Another important finding of this study is the upregulation in Post-CPB biopsies of a gene cluster coding for cell-cycle regulators and mediators of apoptosis. Among them, those coding for DUSP family members, CDKN1A, BTG2, GADD45B,34, and MCL-1 have been previously shown to be upregulated in human myocardial tissues in response to stress and/or DNA damage and to play a role in the regulation of cardiac hypertrophy and remodeling in animal models [74–78]. A cause-effect link between their enhanced expression and CPB has also been suggested [13, 14]. Particularly intriguing is the observation that CPB increased the expression of genes coding for microRNAs (miRs) 21, 22, and 23. miRs are becoming increasingly recognized as key regulators of heart development and function, and altered miR expression has been linked to heart diseases [79, 80], including CHDs [81]. miR-21, miR22, and miR-23 dysregulated levels have been reported to affect cardiac function and to have potential prognostic and/or therapeutic relevance in various cardiovascular disorders [80, 82–85], although conflicting results have indicated a cardioprotective role for miR-21 and miR-22 [86, 87]. A recent report demonstrated changes in the heart miRNome of CHD patients after CPB [88]. However, our study is the first to provide specific evidence of miR-21, miR-22, and miR-23 upregulation in the post-CPB myocardium, identifying new potential molecular biomarkers and therapeutic targets for future investigations in CHDs.

Finally, the expression pattern of genes functionally implicated in cell adhesion and cytoskeleton/matrix organization was also found modulated by CPB, further confirming activation of a remodelling response within the post-CPB myocardium. Among them, CYR61 and ADAMTS1 upregulation was shared by the two groups of patients. The secreted matricellular CYR61 protein was previously found highly expressed in remodeling atrial cardiomyocytes after myocardial infarction and proposed as an early prognostic biomarker of cardiac injury [89], while its mutations have been associated with ASD [90]. ADAMTS1 protein is a metalloprotease induced in the early phase of acute myocardial infarction playing an essential role in the repair of infarcted tissue and the development of heart fibrosis [91, 92]. Other genes selectively upregulated in the ToF myocardium, such as ABRA, XIRP1, THBS1, and HB-EGF, have been previously shown to be associated with pathological cardiac phenotypes [93–95]. Conversely, a few adhesion-related genes were inhibited in ASD atrial samples, among which PRG4 has been found downregulated in the post-CPB ventricular trancriptome [49]. Collectively, our data provide the first evidence that CPB targets different genes involved in cell adhesion/ECM regulation in ToF and ADS, improving our understanding of the mechanisms contributing to cardiac dysfunction after surgery with CPB.

## Conclusion

In conclusion, results from this study provide a better understanding of the molecular pathways specifically involved in ToF and ASD pathogenesis and mediating myocardium response to CPB, demonstrating that gene expression profiling can differentiate these two major forms of CHDs and complement ongoing biomarker development efforts. Early discrimination of patients that may develop intraoperative inflammatory and stress response and associated organ damage is critical to direct tailored post-surgical treatment strategies aimed at minimizing morbid effects of CPB. Our data have important translational value because they enable the identification of candidate genes/pathways that might serve as potential biomarkers of inflammatory response, oxidative stress, and myocardial damage, instrumental for a better prediction of patient prognosis after CPB, as well as possible targets for guiding the development of new specific cardioprotective modalities of intervention in these diseases, that may result in more effective patient management after corrective surgery. In this regard, the demonstration of increased expression of genes encoding critical mediators of myocardial inflammatory injury, such as proinflammatory chemokines, SOCS3, and PTGS2, both in ToF and ASD after CPB is of particular relevance, suggesting that the development of therapeutic approaches that target these genes may be effective in controlling the inflammatory response triggered by CPB in patients affected by different CHDs. On the other hand, it is conceivable that targeting transcription factor-coding genes implicated in the induction of heart hypertrophy, fibrosis, and dysfunction, such as IRF1 KLF10, Bcl3, SOX9, and CEBPB, may represent a new therapeutic opportunity to reduce CPB-dependent damage to cardiac tissues specifically in ToF patients, given their selective upregulation in the ToF myocardium. Hypoxia-associated transcription factors, such as EGR1, may also be regarded as potentially promising therapeutic targets to limit hypoxia pathogenic effects both before (ToF) and after (ToF and ASD) CPB. We acknowledge that the study has some limitations, such as the relatively small sample size analyzed and the fact that patients have not been followed postoperatively to allow evaluation of clinical outcome. Independent experimental validation of the observed transcriptomic patterns in a larger cohort of patients is certainly needed to confirm their prognostic relevance and lead to the development of targeted therapies aimed at reducing the risk of postoperative complications and organ dysfunction after CPB in ToF and ASD.

## Supplementary information

**Additional file 1: Table S1.** Differentially expressed probe sets in TOF vs ASD right atrial samples.

**Additional file 2: Figure S1.** qRT-PCR validation of genes selected from the microarray profile.

**Additional file 3: Table S2.** Differentially expressed probe sets in Post-CPB vs Pre-CPB right atrial samples from ToF patients.

**Additional file 4: Table S3.** Differentially expressed probe sets in Post-CPB vs Pre-CPB right atrial samples from ASD patients.

## Data Availability

The datasets generated and analyzed during the current study are available in the GEO public repository at NCBI (http://www.ncbi.nlm.nih.gov) and can be accessed to through GEO Series accession number GSE132176.

## Abbreviations

CHDs: congenital heart diseases
ToF: Tetralogy of Fallot
ASD: Atrial Septal Defect
CPB: cardiopulmonary bypass
CA: cardioplegic arrest
AoXCL: aortic cross-clamping
I/R: ischemia/reperfusion
ROS: reactive oxygen species
FDR: false discovery rate
FC: fold change
CV: coefficient of variation
DEGs: differentially expressed genes
GEO: Gene Expression Omnibus
GO: gene ontology
GSEA: Gene Set Enrichment Analysis
MSigDB: Broad Institute Molecular Signature v5 Database
miRs: microRNAs
