# Supplementary material for: Transcriptome analysis defines myocardium gene signatures in children with ToF and ASD and reveals disease-specific molecular reprogramming in response to surgery with cardiopulmonary bypass
Source: J Transl Med. 2020 Jan 10;18:21. doi: 10.1186/s12967-020-02210-5 (PMC6954611; doi:10.1186/s12967-020-02210-5)
Supplement: Supplementary file 2 — Additional file 2: Figure S1. qRT-PCR validation of genes selected from the microarray profile. [file 12967_2020_2210_MOESM2_ESM.pdf]

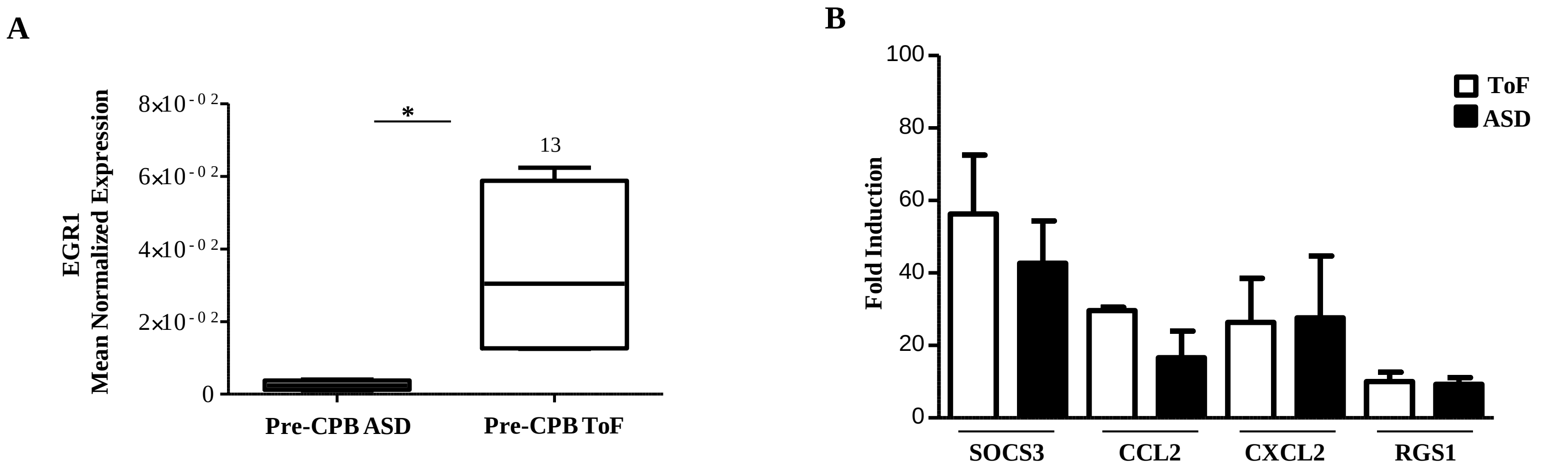

Figure S1. qRT-PCR validation of genes selected from the microarray profile. Expression levels of a subset of genes selected from those listed in Table I and Table III were determined by qRT-PCR analysis in total RNA from ToF and ASD samples profiled by microarray. Expression changes were evaluated in relation to the values obtained for three reference genes, as detailed in the *Methods.* (A) EGR1 transcript levels were compared in atrium tissues from 6 ToF and 6 ADS patients before CPB. Results are shown as a box plot and expressed as mean normalized gene expression values. Boxes show the values falling between the 25th and 75th percentiles, horizontal lines represent mean values, and whiskers (lines that extend from the boxes) represent the highest and lowest values for each group. *p* values of ToF relative to ASD: * $p<0.05$ (unpaired-two-tailed Student't test). Fold-change value in ToF relative to ADS samples is indicated. (B) SOCS3, CCL2, CXCL2, and RGS1 mRNA levels were evaluated in 3 ToF and 3 ASD samples before and after CPB, and relative transcript expression was calculated. Results are expressed as mean fold-increase ± SD of three determinations/donors in post-CPB relative to pre-CPB samples.
